# Mass spectrometry of intact membrane proteins: shifting towards a more native-like context

**DOI:** 10.1042/EBC20220169

**Published:** 2023-03-29

**Authors:** Abraham Oluwole, Denis Shutin, Jani R. Bolla

**Affiliations:** 1 Department of Chemistry, University of Oxford, South Parks Road, Oxford OX1 3QZ, U.K.; 2 The Kavli Institute for Nanoscience Discovery, Oxford, South Parks Road, Oxford OX1 3QU, U.K.; 3 Department of Biology, University of Oxford, South Parks Road, Oxford OX1 3RB, U.K.

**Keywords:** lipids, membrane proteins, native mass spectrometry, structural biology

## Abstract

Integral membrane proteins are involved in a plethora of biological processes including cellular signalling, molecular transport, and catalysis. Many of these functions are mediated by non-covalent interactions with other proteins, substrates, metabolites, and surrounding lipids. Uncovering such interactions and deciphering their effect on protein activity is essential for understanding the regulatory mechanisms underlying integral membrane protein function. However, the detection of such dynamic complexes has proven to be challenging using traditional approaches in structural biology. Native mass spectrometry has emerged as a powerful technique for the structural characterisation of membrane proteins and their complexes, enabling the detection and identification of protein-binding partners. In this review, we discuss recent native mass spectrometry-based studies that have characterised non-covalent interactions of membrane proteins in the presence of detergents or membrane mimetics. We additionally highlight recent progress towards the study of membrane proteins within native membranes and provide our perspective on how these could be combined with recent developments in instrumentation to investigate increasingly complex biomolecular systems.

## Introduction

Integral membrane proteins comprise approximately one-third of the human proteome [1] and undertake key roles in signal and energy transduction, cell adhesion, molecular transport, and enzymatic catalysis [2]. Such functions are carefully regulated by post-translational modifications and biomolecular interactions that fine-tune protein activity in response to internal and external stimuli. When these mechanisms fail, the subsequent dysregulation of membrane protein function can induce a wide range of pathological conditions including cardiovascular, central nervous system, and immune disorders. Accordingly, certain classes of membrane proteins (e.g. G-protein coupled receptors [GPCRs] and ion channels) have been extensively employed as biological targets within drug discovery programs investigating such indications [3–5].

Understanding how endogenous binding partners and drugs impact the structures and function of membrane proteins is a key step towards elucidating protein structure–function relationships. Advances in experimental and computational techniques have enabled access to hitherto elusive knowledge of membrane proteins’ working mechanisms and their interactions with other biomolecules. These methodological advancements range from developments in membrane protein crystallisation [6] and solubilisation methods [7] to high-resolution cryo-electron microscopy [8] and tools for predicting protein structure [9,10]. In parallel, native mass spectrometry (native MS) has emerged as a powerful technique that can provide unique insights [11–16]. Native MS analysis of purified membrane proteins provides direct information regarding purity, folding, modifications, oligomeric state, and sub-unit interactions in homo- or heterooligomers. Owing to the possibility of monitoring protein-ligand interactions with molecular-level precision, native MS has developed into a valuable tool for high-throughput drug screening platforms [17,18].

By integrating native MS alongside other structural techniques, deep insights into the relationship between protein sequence, structure, dynamics, and function can be derived. The ease of coupling native MS with ion-mobility and hydrogen-deuterium exchange MS enables access to information regarding protein folding, conformation, and dynamics [19–23]. Similarly, combining native MS directly with cryo-EM via selective deposition using electrospray ionisation offers additional benefits of increasing the scope and throughput of structural determination [24–26]. Native MS can serve as a preliminary tool to screen for the best protein homologues and optimal purification conditions for subsequent interrogation by other structural techniques [27,28]. Additionally, native MS enables the detection of lipids bound to membrane proteins [29,30] – offering valuable insights into the critical role of lipids in membrane protein structures and functions.

There has been a multitude of excellent, in-depth reviews focused on the study of membrane protein structure by native MS [15,31–33]. Herein we focus on recent studies investigating interactions between membrane proteins and endogenous ligands captured throughout the purification process and the dynamics of membrane proteins in signalling pathways observed directly in the native membrane. Finally, we will draw attention to the native top-down MS approach that combines native MS with top-down proteomics in a single experiment and give our perspective on how native top-down can be used to study complex signalling pathways in native membranes.

## Native mass spectrometry of detergent-purified and detergent-free membrane proteins

A fundamental challenge associated with handling membrane proteins outside of their cellular context is to maintain their native fold in aqueous buffers. This is often achieved using detergents, which mimic the phospholipid bilayer [34,35]. Detergents can drastically impact the structure and function of solubilised membrane proteins [36], and an optimal detergent for a biophysical technique must be determined on a case-by-case basis [37]. Fortunately, there exists a wide range of detergents with varying properties to select from, with both commercial and specialist detergents offering assorted head groups and chain chemistries. Many detergents typically used to maintain protein stability and function are not always suitable for native MS measurements. For example, DDM (*n*-dodecyl β-D-maltoside) and LMNG (lauryl maltose neopentyl glycol) are often considered the preferred detergents for protein extraction and purification from cellular membranes as they create a stable environment for proteins [38]. However, these surfactants form relatively larger micelles which can only be removed from the protein in the gas phase using extensive collisional activation [39,40]. Therefore, when using DDM or LMNG in native MS measurements, it can be difficult to liberate the protein from the micelle without inducing ligand and/or subunit dissociation. By contrast, native MS-friendly detergents such as the charge-reducing C8E4 and LDAO, considered to provide a harsher environment for proteins, can be removed at lower collisional energies, assisting in the preservation of non-covalent interactions between membrane proteins and their binding partners. However, such detergents may be less effective at maintaining the protein’s fold in solution. There is a delicate balance between activation energies and preserving interactions of membrane proteins in native MS analyses, so care must be taken in selecting the detergents for MS analyses [41,42]. Table 1 exemplifies the variety of detergents used for protein solubilisation and subsequent native MS analyses over the past 3 years. We hope that a future database presenting ‘reference’ mass spectra of membrane proteins characterised to date, along with suitable experimental conditions and instrument settings can guide the community in selecting optimal detergents suitable for new protein targets.

**Table 1:** Selected list of membrane proteins purified in various detergents and analysed by native MS in recent years (2020–2022)

Membrane protein	Detergent used for purification	Detergent used for native MS analysis	References
**2022**			
Kirbac3.1	DDM	C8E4	[79]
Aqp0		C8E4	[12]
Aqp0	OG	C8E4	[56]
OmpF, FepA, BtuB	OG	OG	[65]
TqsA	DDM	LDAO, C8E4	[80]
FhaC	OG	OG	[81]
GCGR	DDM, CHS	OGDs G1	[82]
Glut5 PfHT1 GlpG NTR1	DDM DDM DDM LMNG	DDM DDM DDM, LDAO DDM, NM, DDTM, LDAO, C8E4, C12E8, LMNG, OGNG, Fos-choline-12, -14, -16, HEGA-11, MEGA-10, sucrose monodecanoate	[83]
NHA2	DDM, CHS	C12E9	[84]
β1AR	DDM	DDM/Foscholine16/cholesteryl hemisuccinate	[85]
MraY MurJ	DDM	DDM, OG, C8E4, LDAO, LDAO	[48]
NIP2;1	DDM, DM	LDAO	[86]
PA2880	DDM	DDM	[87]
PatAB	DDM	OGD G1	[88]
PMP22	Empigen BB, DM	C12E9	[59]
Rho and opsin	LMNG	LMNG	[54]
SthK	DDM	OGNG	[89]
TRAAK TREK2	C10E5	C10E5	[90]
**2021**			
A2aR GCGR	LMNG G1, CHS	LMNG G1/CHS	[72]
AKCR1	LMNG, CHA	DDM	[91]
BAM complex	DDM	C8E4	[68]
bR, AR3	OG	OG	[30]
Kir3.4 Kir3.2	C10E5	C10E5	[92]
LptDE	DDM	C8E4	[67]
MFSD2A	DDM, CHS	DDM	[93]
RAGE	Triton X-100	Triton X-100	[94]
Rhodopsin	CHAPS	C8E4	[95]
SusCD	LDAO	C8E4	[74]
TmrAB	DDM	DDM	[96]
TolC	OG	OG	[97]
tOmpA, OmpAAG, OmpTrans2, OmpTrans3	DDM	DDM	[98]
Wzc	LMNG	C8E4, LDAO	[57]
**2020**			
AceI	DDM	LDAO, C8E4, DDM, OGNG	[66]
AcrB	DDM	DDM, Triton X-100	[99]
AmtB	DDM	OGD	[100]
AqpZ MATE AmtB BAM NTSR1 OmpT	DDM and OGDs	OGDs	[43]
CD9, CD81	DDM	DDM	[101]
Cytochrome bc1 complex	GDN	GDN	[102]
DGAT1			[103]
EmbB	DDM	C12E8, DDM	[104]
FapF, FapD	C8E4	C8E4	[76]
FptA	OG	OG	[105]
Get1/2	C12E9, Cymal-5/6, DM, UDM, DDM, LMNG, Cymal-5-NG	UDM, LMNG, C12E9, Cymal-5-NG	[106]
HAS	DM	DM	[107]
K2p4.1	C10E5	C10E5	[108]
Lci1	OG	LDAO	[109]
NHE9	DDM	DDM	[110]
OmpF	OG	OG	[111]
PSH LeuT MurJ	NG, LDAO DDM DDM	NG, LDAO OG LDAO	[66]
Semisweet DHODH CB1 Beta-1 AR OmpF AqpZ AmtB TSPO	DDM LDAO LMNG DDM OG DDM DDM DDM	C8E4 LDAO DDM, foscholine, CHS DDM, foscholine, CHS C8E4 C8E4 C8E4 C8E4	[78]
V-ATPase	DDM	LMNG	[60]

Excitingly, new detergents with modified headgroups, linkers, and chain configurations continue to emerge. A notable example in this regard is the development of a ‘tailored’ OGD-based detergent that combines the membrane solubilisation and protein extraction efficacy of DDM with the ease of protein release from C8E4 micelles [43]. Synthesis of such hybrid detergents containing two different headgroups [44] can foster the creation of detergents with predictable biophysical properties, paving the way for an ideal detergent tailored towards a given membrane protein target and downstream analysis technique. Nevertheless, an optimal detergent for solubilising and stabilising a given membrane protein target is currently difficult to predict.

Detergent-purified proteins can also be reconstituted into other membrane mimetics, such as bicelles, amphiphilic polymers, liposomes and nanodiscs, for native MS analyses [45,46]. However, the initial exposure to detergents may impact the observed oligomeric state of the protein, as well as strip away structurally or functionally important endogenous lipids and ligands. In those cases, adopting a completely ‘detergent-free’ strategy is desirable. To this end, membrane proteins can be directly solubilised by synthetic polymers, including SMA and DIBMA, forming the so-called native nanodiscs [47,48]. This detergent-free strategy is highly appealing since the protein is maintained in a lipid bilayer composed of its own native lipids. However, releasing intact protein complexes from SMALPs is nontrivial, potentially due to low charge acquisition by proteins embedded in the nanodiscs [49], the heterogeneity of the system, and strong interactions between the protein–lipid complex and the scaffold polymer. Over the past decade, significant efforts have been dedicated to optimising the protocols to eject samples from these membrane mimetics and in several instances, it was possible to capture protein-lipid interactions at even lower activation energies than those used to remove detergent micelles [33]. Additionally, selection of the appropriate membrane mimetic can have a significant impact on the observed results; for example, the proportion of PMP22 dimer was shown to be different in detergent micelles or when reconstituted into SCOR bicelles that better mimic the cholesterol and sphingolipid-rich native membrane environment (PMP22 is further discussed in a later section) [50]. Nanodiscs, that consist of a lipid bilayer surrounded by membrane scaffolding protein, have also been gaining popularity recently. Using nanodiscs with a 50:50 distribution of two different lipids allowed the investigation of lipid selectivity for two membrane proteins, AmtB and AqpZ [51]. AqpZ displayed the preference for PG over PE, and PC over either lipid; while AmtB was shown to be broadly enriched in bound PG, with a few tightly bound PC molecules. More complex mixed lipid nanodiscs that mimic various biological membranes were also recently investigated [52]. In combination with methodological improvements, such as controlling the stability of membrane protein nanodiscs by adding charge manipulation reagents [53], these developments could lead to even more widespread use of nanodiscs in native MS in the near future. Nevertheless, the possibility of scrutinising membrane proteins directly solubilised and purified in the complete absence of traditional detergents continues to gain interest. For example, a recent study demonstrates that SMA copolymer can selectively solubilise and maintain bacteriorhodopsin and archeerrhodopsin-3 in a correctly folded conformation, unlike detergents [30]. Further method developments, such as supercharging or new activation methods, can further improve the analysis of SMALP-solubilised membrane proteins. An exciting detergent-free method has recently emerged that involves the ejection of lipid vesicles derived directly from native membranes [54,55]; this is discussed in more detail in a later section.

## Probing covalent modifications and non-covalent interactions of membrane proteins

Although the use of native MS in probing the interactions of membrane proteins has been well described in recent reviews [32,33], here we highlight the recent observations made in the past 3 years.

### Covalent modifications

While native MS (not coupled with doing multiple rounds of MS/MS), does not provide localisation information on covalent modifications to proteins, it can still play a role in the characterisation of individual proteoforms. Such measurements can enable the effects of PTMs on protein interactions to be directly identified. For example, Harvey et al. [56] employed native MS to study post-translational modifications of AQP0, the most abundant membrane protein in the eye lens. By performing surface-induced dissociation on doubly phosphorylated tetramers, they discovered that these forms contain exclusively singly phosphorylated monomers, despite the presence of two phosphorylation sites on each monomer. In a different study, native MS was used to monitor the number of phosphorylations on Wzc, a protein involved in EPS regulation in Gram-negative bacteria, to complement high-resolution structural studies [57]. The non-phosphorylated form of the protein assembled into octamers while the multiply phosphorylated form was monomeric, indicating a potential regulatory mechanism for glycan polymerisation and translocation.

On the other hand, coupling native MS with tandem MS (MS^n^), known as native top-down MS, can enable the identification and characterisation of specific proteoforms and post-translational modifications. In an elegant study, Ro et al. [58] recently coupled nanodiscs with native top-down MS and gained valuable insights into the active form of particulate methane monooxygenase, pMMO by determining the stoichiometry of copper ions bound to each subunit and detecting post-translational modifications.

### Non-covalent interactions

#### Protein–protein

A more common application of native MS is in the investigation of non-covalent biomolecular interactions, such as protein–protein interactions. Fantin et al. [59] compared the oligomerisation of PMP22 between the wild-type protein and disease-associated mutants and detected decreased proportions of dimer for mild and moderate disease phenotypes but enhanced dimerisation for the severe disease mutants. By employing ion-mobility MS, the authors observed greater gas-phase stability of dimers but lower stability of monomers of all mutants compared with the wild-type protein with respect to unfolding. This led to a proposed pathophysiological mechanism in which disease-associated mutants are more likely to aggregate, resulting in extensive mistrafficking. As another example, native MS has been extremely useful for confirming the subunit composition of the V1 region of the mammalian V-ATPase [60].

#### Protein–lipid

**Figure 1:**
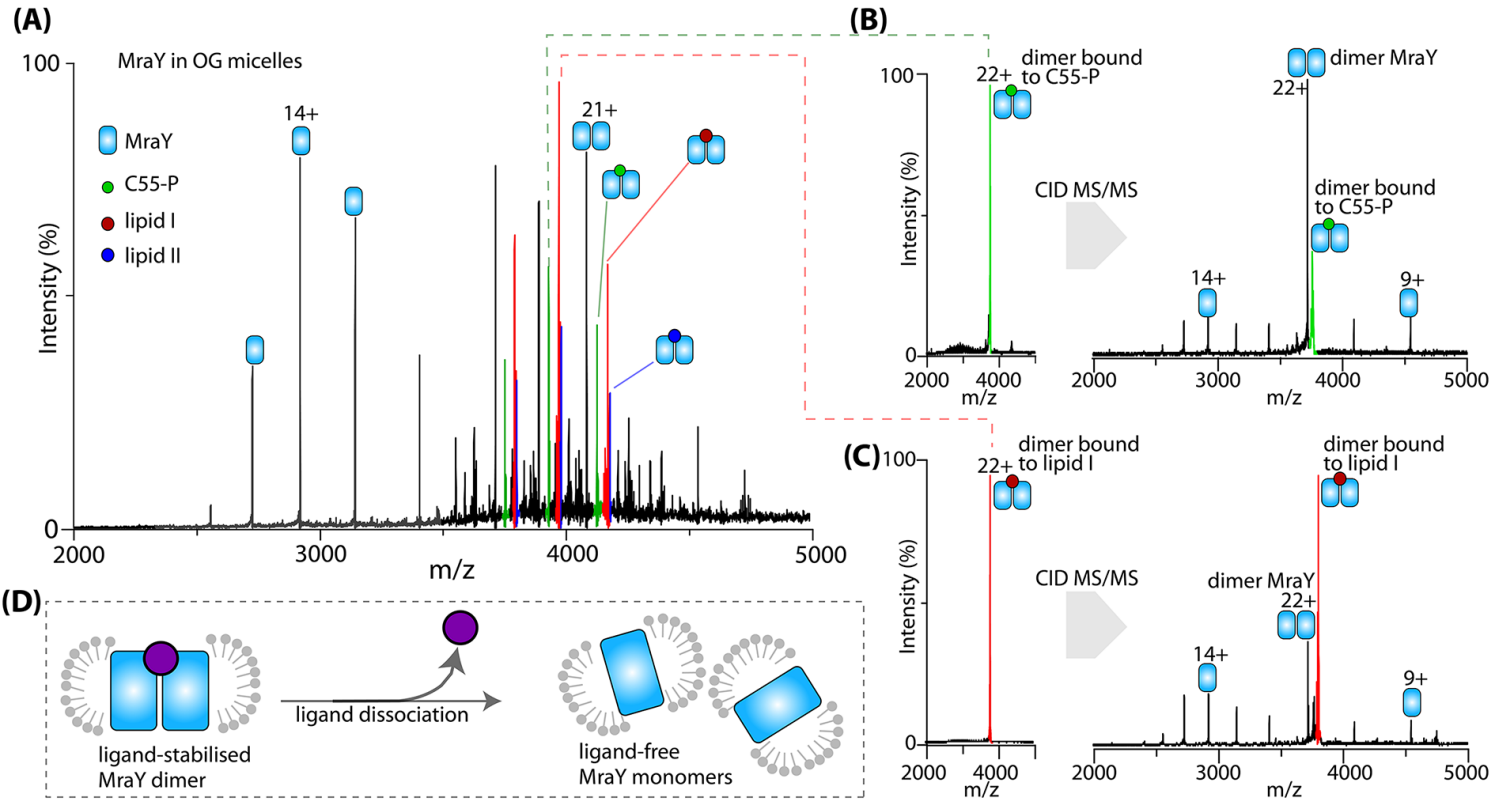
Native mass spectrometry of purified MraY (**A**) Mass spectrum of MraY released from OG (octylglucoside) detergent micelles. Peaks in the spectrum correspond to the MraY monomer, the ligand-free dimer, and the dimer bound to undecaprenyl phosphate (C55P), lipid I and lipid II. (**B** and **C**) Dimer-bound to C55P (B) and lipid I (C) were separately isolated and subjected to collision-induced dissociation (CID) to liberate MraY protomers. (**D**) Schematic depiction of a ligand stabilised MraY dimer in detergent micelles. Removal of bound ligand caused the dimer to dissociate. Data reproduced from [63].

Lipids can influence the structure and function of membrane proteins. For example, while some oligomeric membrane proteins contain strongly bound inter-subunit interfaces, other weakly oligomerise and readily dissociate into monomers or lower oligomeric forms upon extraction from their native lipid environment. It has been suggested [61] that in these cases, specific lipid interactions may be required for oligomeric assembly in the phospholipid bilayer. One approach to elucidate the effect of lipids on membrane proteins is to titrate a detergent-purified, fully delipidated sample with the most abundant lipids in their native membrane and quantify the relative binding affinities [62]. The specificity of bound ligands can then be ascertained by systematic titration with a delipidating detergent such as NG as demonstrated for the lipid II flippase MurJ [41]. In another study, MraY dimer, the first integral membrane enzyme in the biosynthesis of bacterial cell wall peptidoglycan, was found to be stabilised by endogenous lipids [63]. Further investigation by MS/MS and functional assays revealed the identity of these ligands as substrate and product of MraY-catalysed reaction (Figure 1). Furthermore, Patil et al. [64] found densities at the cavity formed by the transmembrane helices of human GPR158. PE and PI lipids were identified by native MS and lipidomics and remodelled into the EM structure with high confidence and their roles were ascribed to stabilising the dimer interface. More recently, native MS combined with chemical crosslinking was used to probe the organisation of bacterial outer membrane proteins and lipids [65]. The pattern of lipid crosslinks captured by native MS amongst various OmpF mutants reflects that the outermost leaflet of the outer membrane is enriched with lipopolysaccharides while the inner leaflets are mainly composed of phospholipids.

#### Protein–drug/antibiotics

Another interesting area of research is the interaction of membrane proteins with drugs or antibiotics. Using native MS, the antibiotic chlorhexidine was shown to induce a change in the oligomeric state of the chlorhexidine efflux pump AceI [66]. Chlorhexidine was also shown to cause an increase in the formation of the functional dimeric form of AceI. In 2021, Fiorentino et al. [67] investigated the mechanism by which an antibacterial peptide thanatin impairs LPS translocation through LptDE complex. Native MS confirmed the simultaneous binding of thanatin and LPS to LptDE, ruling out inhibition by competitive binding. This result, in combination with hydrogen-deuterium exchange MS and molecular dynamics simulations data, suggested a mechanism whereby thanatin obstructs LPS transport by stabilising the inactive form of LptDE. In another study, a combination of native MS and MD simulations enabled the elucidation of the lipid-binding preference of the Bam complex [68]. The study revealed that the Bam complex preferentially binds cardiolipin and that the latter enhances its interaction with darobactin, a peptidomimetic antibiotic.

#### Protein–metal ion

One type of interaction that is challenging to observe by native MS is the binding of proteins to non-volatile salt ions, such as Na^+^. Such ions have been previously shown to regulate the assembly of several membrane protein complexes [69]. In native MS, the presence of even relatively low concentrations of salts results in signal suppression, loss of resolution and spectra that are often too difficult to interpret. Consequently, native MS typically employs volatile salts, such as ammonium acetate, that are readily removed upon gentle activation [70]. Nevertheless, there are instances where metal ions are necessary cofactors for protein stability and enzymatic reactions. One way to study the effect of non-volatile salts on protein structure and stability is to use emitters with submicron tip diameters that form smaller electrospray droplets and thus greatly reduce the number of observed protein–salt adducts [71]. This approach was recently used by [72] to study the effects of sodium ions on two GPCRs: a Class A GPCR, A2aR, and a Class B GPCR, GCGR. For A2aR, native MS revealed enhanced Na^+^ binding in the presence of antagonists and suppressed Na^+^ binding in the presence of agonists. This result is consistent with previous structural studies that showed the presence of an allosteric Na+ binding pocket in inactive conformations of Class A GPCRs, which is absent in the active conformations. For GCGR, a more stable conformation was observed in NaCl/Tris buffer compared to ammonium acetate, as indicated by the lower charge state distribution and enhanced binding of the stabilising negative allosteric modulator, NNC0666, observed in the former buffer. These results highlight the applicability of native MS to study membrane proteins from physiologically relevant salt buffers. More recently, another native MS approach was developed to interrogate the propensity of β_1_-adrenergic receptor coupling to G_s_ and G_i_. Upon capturing the G-protein-coupling complexes in presence of different ligands, it was observed that endogenous zinc ions specifically stabilise the complexes between G-proteins and receptors. Overall, this study uncovered a novel role for metal ions in modulating the selectivity of G-protein coupling [73].

#### Protein–ligand/Enzyme–substrate

To probe the interactions with ligands, in one study Gray et al. [74] combined native MS analysis with other structural tools to identify the finite size of the substrate molecules (approximately 2.5 kDa) that are imported by the SusCD complex, a major fructooligosaccharides uptake system in Bacteroidetes. Native MS was also used to study the interactions of membrane proteins, involved in peptidoglycan biosynthesis pathway, with lipid-like substrates. The key step in the synthesis of peptidoglycan is the translocation of lipid II from the inner to the outer leaflet of the plasma membrane. The flippase MurJ is thought to play a dominant role over FtsW in this process. A previous native MS study revealed a stronger lipid II binding preference for MurJ over FtsW, and that the lipid II binding to MurJ is reduced in the presence of cardiolipins, which is suggestive of lipid-regulated precursor translocation [75]. Similarly, an increase in the binding affinities of MraY towards C55P, MurG towards lipid I, and MurJ towards lipid II were observed using native MS recently, suggesting that the affinity of each membrane enzyme for its native substrate promotes their trajectory along the peptidoglycan pathway [63].

## Transferring membrane proteins into the mass spectrometer: a shift from isolated solubilised protein target towards endogenous proteoform complexes within their native membrane context

When using even the most ‘native-like’ membrane mimetic, there is always a risk of experimental artefacts caused by the extraction of the protein from its native membrane environment. Recently, membrane proteins and their complexes have been released into the mass spectrometer directly from their native membrane, marking a significant shift towards native MS of membrane proteins in the most native-like contexts. In 2018, Chorev et al. [55] used native MS to investigate protein complexes directly from native membrane vesicles. This technique named sonicated lipid vesicles MS (SoLVe MS) was successful in revealing a range of subunit interactions and lipid preferences for endogenous proteins that are abundant in the membrane. Several of the observed complexes were not previously reported, highlighting the potential of SoLVe MS. For example, the Bam complex with either a second BamE subunit or up to three cardiolipin molecules was observed, providing more structural insight about this complex. The interactions between SecYEG translocon and an F-type ATPase were also observed directly.

**Figure 2:**
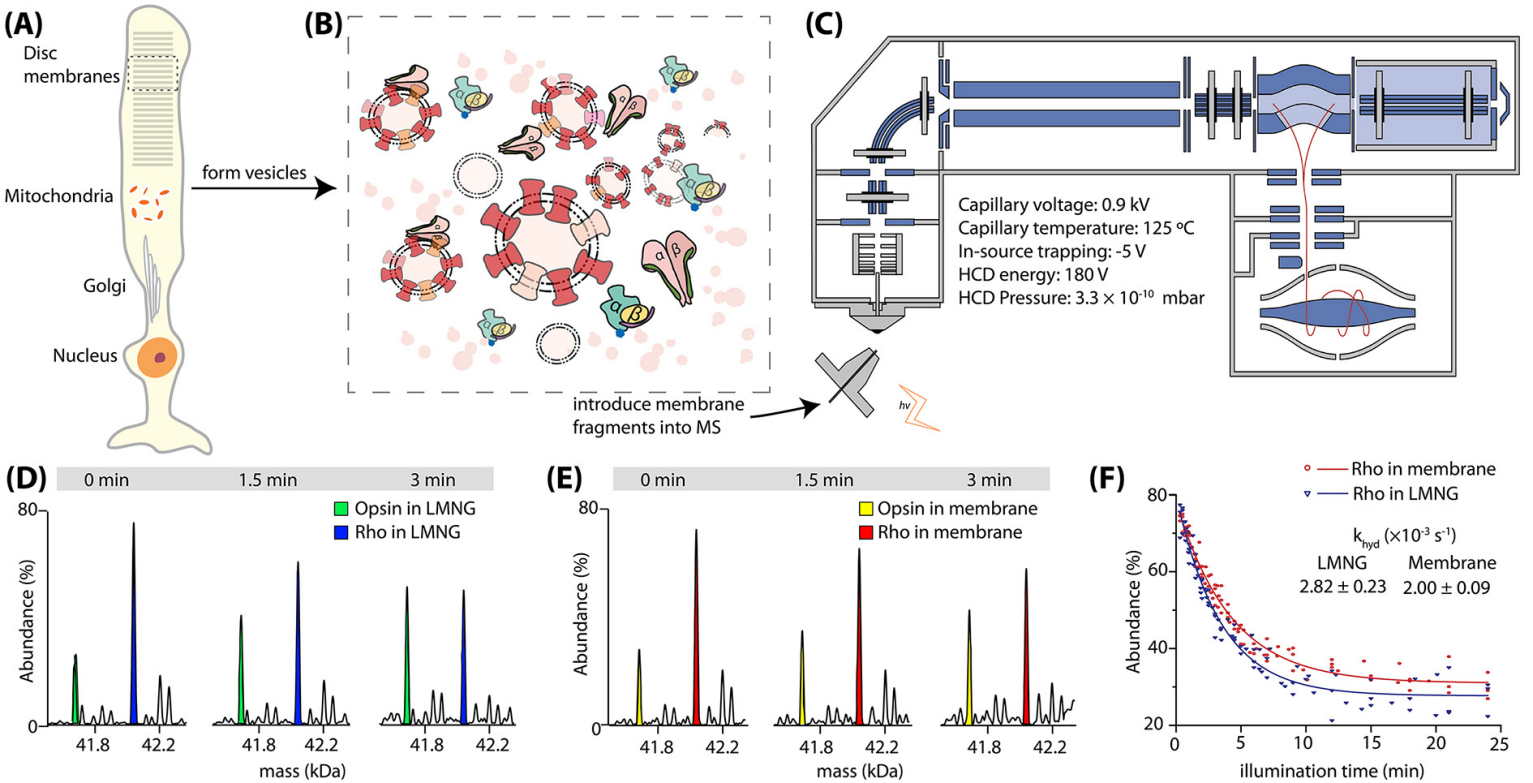
Rhodopsin signalling cascade in the native membrane captured by mass spectrometry (**A–C**) Disc membranes of rod cells (A) are homogenised to form a heterogeneous distribution of vesicles (B) that are introduced directly into the mass spectrometer (C), a UHMR equipped with an LED light source. (**D,E**) Changes in the population of rho and opsin in LMNG (blue and green, respectively) and in the native membrane vesicles (red and yellow, respectively; Individual spectra are shown as zero-charge plots with illumination times stated. (**F**) Relative abundance of rho as a function of illumination time in LMNG micelles (blue) or native membranes (red). Figures reproduced from [54].

In a very recent study, Chen et al. [54] ejected retinal rod segments (ROSs) directly from the native bovine disc membrane into the mass spectrometer under controlled light conditions to uncover signalling events of a GPCR (rhodopsin) in the context of its own native membrane (Figure 2). Crucially, all components of the signalling pathway were observed in real-time and without any extensive sample pre-treatments. The study found that one of the key steps in the pathway, the hydrolysis of retinal, was significantly slower in native membranes compared to LMNG detergent micelle. In addition, the presence of PE lipids in ROS membranes was shown to be important for the regeneration of rho species following the original activation by light. Unsaturated PC lipids were observed to be enriched around rho shortly after light activation, shedding light on the role of lipid microenvironments for signalling facilitation. The ability to observe all the steps of the signalling pathway directly from the native membranes enabled kinetic studies in the presence of exogenously added downstream effector molecules, highlighting the potential application of this methodology in drug discovery workflows.

Despite the numerous advantages and a recently published protocol [76], the practical application of SoLVe MS remains challenging. The main obstacle is that the identities of various complexes observed in the spectrum are difficult to achieve through mass alone. Various other techniques exist that can be combined with native MS to further characterise species observed in SoLVe MS spectra. Native top-down mass spectrometry employing high energy collision- (CID HCD), electron- (ECD, ETD and EID) and photon- (IRMPD and UVPD) based activation techniques can be used to break covalent bonds (while preserving non-covalent interactions in some cases) to obtain information about protein sequence and identify proteoforms. Recent advances in this field have been outlined in a recent review [31]. In addition, co-purified lipids and other small molecules often need to be identified. A traditional approach to this problem is to perform lipidomics on the co-purified lipids in a separate experiment to native MS [77]; however, while this method can be very informative, the direct protein–lipid binding information is lost during the extraction step. The recent advances in MS instrumentation enabled the development of a hybrid method termed ‘nativeomics’ (also known as native top-down), this allows observation and identification of protein-bound lipids and ligands in a single experiment [78]. For instance, the application of this workflow to an outer membrane protein TSPO allowed previously unidentified electron density in the crystal structure of this protein to be assigned as PE lipid species [78].

## Future perspectives and concluding remarks

Native MS is a versatile and rapid technique for obtaining non-covalent interactions of membrane proteins that are often difficult or outright intractable to other techniques. It can be used to study membrane proteins in a varied range of membrane mimetic systems, notably in their own native lipid bilayer. Advances in the synthesis and commercialisation of mild and native MS-compatible detergents will continue to be relevant for investigating protein–drug/lipid binding where highly purified samples are required. Increasing the repertoire of activation regimes that enables release of intact oligomers from lipid bilayer nanodiscs will allow selective disruptions of the nanodiscs with minimal dissociation of the encapsulated protein complexes. Such an advance will pave the way for releasing intact proteins from more complex biological membranes and tissues for which strong lipid–protein interactions must be overcome.

Recent advances in native MS have enabled protein assemblies to be released from intact lens tissue [12]. For such a method to be widely applicable, it will be critical to identify novel sample preparation strategies and develop gas phase activation techniques that ensure efficient but selective dissociation. Tagging of endogenous proteins for enrichment with affinity binders and the development of new affinity probes to enrich the target proteins over other noninteracting members of the proteome will enhance the signal-to-noise ratio in applying this method to investigations of specific pathways. It will be interesting to be able to analyse various proteoforms in a healthy versus diseased state in a single experiment to accelerate diagnosis, monitor disease progression, and perform drug screening in the native context of their protein targets – towards this goal, native top-down holds great promise.

## Summary

- In the modern era of structural biology, native MS has emerged as a powerful tool for defining the molecular sociology of a variety of membrane protein targets including transporters, channels, and receptors. Starting off with detergents and membrane mimetics, this area of research is now going through a transformational period towards more native environments.
- Although still in its infancy, analysis of complexes directly from native membranes by native MS has already managed to capture several key signalling events. However, the full potential and widespread use of this approach are yet to happen.
- In addition to optimising sample preparation strategies, there is a clear need for new instrumentation, which combines sensitivity, dynamic mass range, resolution, and access to diverse ion activation techniques including HCD, ECD, ETD, IRMPD and UVPD.

## Open Access

Open access for this article was enabled by the participation of University of Oxford in an all-inclusive *Read & Publish* agreement with Portland Press and the Biochemical Society under a transformative agreement with JISC.

## Abbreviations

A2aR: adenosine 2A receptor
C8E4: tetraethylene Glycol Monooctyl Ether
CID: collision induced dissociation
Cryo-EM: cryogenic electron microscopy
DDM: *n*-dodecyl β-D-maltoside
DIBMA: diisobutylene-maleic acid
ECD: electron-capture dissociation
EID: electron-induced dissociation
ETD: electron-transfer dissociation
GCGR: glucagon receptor
GPCR: G-protein-coupled receptor
HCD: higher-energy collisional dissociation
HDX: hydrogen-deuterium exchange
IRMPD: infrared multiple photon dissociation
LDAO: *n*-Dodecyl-N,N-Dimethylamine-N-Oxide
LMNG: lauryl maltose neopentyl glycol
MS: mass spectrometry
NG: nonylglucoside
OG: octylglucoside
OGD: oligoglycerol detergents
PC: phosphatidylcholine
PE: phosphatidylethanolamine
SMA: styrene maleic anhydride
SMALPS: styrene-maleic acid lipid particles
SoLVe-MS: sonicated lipid vesicle mass spectrometry
UVPD: ultraviolet photodissociation
XL-MS: cross-linking mass spectrometry

## References

[1] Dobson L., Reményi I. and Tusnády G.E. (2015) The human transmembrane proteome. Biol. Direct 10, 31 10.1186/s13062-015-0061-x 26018427 PMC4445273

[2] Marinko J.T., Huang H., Penn W.D., Capra J.A., Schlebach J.P. and Sanders C.R. (2019) Folding and misfolding of human membrane proteins in health and disease: from single molecules to cellular proteostasis. Chem. Rev. 119, 5537–5606 10.1021/acs.chemrev.8b00532 30608666 PMC6506414

[3] Santos R., Ursu O., Gaulton A., Bento A.P., Donadi R.S., Bologa C.G. et al. (2017) A comprehensive map of molecular drug targets. Nat. Rev. Drug Discovery 16, 19–34 10.1038/nrd.2016.230 27910877 PMC6314433

[4] Sriram K. and Insel P.A. (2018) G protein-coupled receptors as targets for approved drugs: how many targets and how many drugs? Mol. Pharmacol. 93, 251–258 10.1124/mol.117.111062 29298813 PMC5820538

[5] Hutchings C.J., Colussi P. and Clark T.G. (2019) Ion channels as therapeutic antibody targets. MAbs 11, 265–296 10.1080/19420862.2018.1548232 30526315 PMC6380435

[6] Nageswar K.V., Sharma M., Patil D.N., Nayak S., Roy A. and Singh A.K. (2022) Current trends in membrane protein crystallography. Advances in Protein Molecular and Structural Biology Methods, pp. 277–290, Elsevier 10.1016/B978-0-323-90264-9.00018-0

[7] Brown C.J., Trieber C. and Overduin M. (2021) Structural biology of endogenous membrane protein assemblies in native nanodiscs. Curr. Opin. Struct. Biol. 69, 70–77 10.1016/j.sbi.2021.03.008 33915422

[8] Kühlbrandt W. (2022) Forty years in cryoEM of membrane proteins. Microscopy 71, i30–i50 10.1093/jmicro/dfab041 35275191 PMC8855526

[9] Jumper J., Evans R., Pritzel A., Green T., Figurnov M., Ronneberger O. et al. (2021) Highly accurate protein structure prediction with AlphaFold. Nature 596, 583–589 10.1038/s41586-021-03819-2 34265844 PMC8371605

[10] Baek M., DiMaio F., Anishchenko I., Dauparas J., Ovchinnikov S., Lee G.R. et al. (2021) Accurate prediction of protein structures and interactions using a three-track neural network. Science 373, 871–876 10.1126/science.abj8754 34282049 PMC7612213

[11] Barth M. and Schmidt C. (2020) Native mass spectrometry-a valuable tool in structural biology. J. Mass Spectrom. 55, e4578 10.1002/jms.4578 32662584

[12] Hale O.J. and Cooper H.J. (2022) Native ambient mass spectrometry of an intact membrane protein assembly and soluble protein assemblies directly from lens tissue. Angew. Chem. Int. Ed. 61, e202201458 10.1002/anie.202201458 PMC9401010 35665580

[13] Hammerschmid D., van Dyck J.F., Sobott F. and Calabrese A.N. (2020) Interrogating membrane protein structure and lipid interactions by native mass spectrometry. Methods Mol. Biol. 2168, 233–261 10.1007/978-1-0716-0724-4_11 33582995

[14] Jooß K., McGee J.P. and Kelleher N.L. (2022) Native mass spectrometry at the convergence of structural biology and compositional proteomics. Acc. Chem. Res. 55, 1928–1937 10.1021/acs.accounts.2c00216 35749283 PMC9296611

[15] Karch K.R., Snyder D.T., Harvey S.R. and Wysocki V.H. (2022) Native mass spectrometry: recent progress and remaining challenges. Annu. Rev. Biophys. 51, 157–179 10.1146/annurev-biophys-092721-085421 34982572 PMC10700022

[16] Bennett J.L., Nguyen G.T. and Donald W.A. (2021) Protein–small molecule interactions in native mass spectrometry. Chem. Rev. 122, 7327–7385 10.1021/acs.chemrev.1c00293 34449207

[17] Gavriilidou A.F., Sokratous K., Yen H.-Y. and De Colibus L. (2022) High-throughput native mass spectrometry screening in drug discovery. Front. Mol. Biosci. 9, 837901 10.3389/fmolb.2022.837901 35495635 PMC9047894

[18] Reher R., Aron A.T., Fajtová P., Stincone P., Wagner B., Pérez-Lorente A.I. et al. (2022) Native metabolomics identifies the rivulariapeptolide family of protease inhibitors. Nat. Commun. 13, 4619 10.1038/s41467-022-32016-6 35941113 PMC9358669

[19] Dafun A.S. and Marcoux J. (2022) Structural mass spectrometry of membrane proteins. Biochim. Biophys. Acta 1870, 140813 10.1016/j.bbapap.2022.140813 35750312

[20] Giladi M. and Khananshvili D. (2020) Hydrogen-deuterium exchange mass-spectrometry of secondary active transporters: from structural dynamics to molecular mechanisms. Front. Pharmacol. 11, 70 10.3389/fphar.2020.00070 32140107 PMC7042309

[21] Martens C. and Politis A. (2020) A glimpse into the molecular mechanism of integral membrane proteins through hydrogen–deuterium exchange mass spectrometry. Protein Sci. 29, 1285–1301 10.1002/pro.3853 32170968 PMC7255514

[22] Shukla A.K., Westfield G.H., Xiao K., Reis R.I., Huang L.-Y., Tripathi-Shukla P. et al. (2014) Visualization of arrestin recruitment by a G-protein-coupled receptor. Nature 512, 218–222 10.1038/nature13430 25043026 PMC4134437

[23] Jia R., Martens C., Shekhar M., Pant S., Pellowe G.A., Lau A.M. et al. (2020) Hydrogen-deuterium exchange mass spectrometry captures distinct dynamics upon substrate and inhibitor binding to a transporter. Nat. Commun. 11, 6162 10.1038/s41467-020-20032-3 33268777 PMC7710758

[24] Mikhailov V.A., Mize T.H., Benesch J.L. and Robinson C.V. (2014) Mass-selective soft-landing of protein assemblies with controlled landing energies. Anal. Chem. 86, 8321–8328 10.1021/ac5018327 25026391

[25] Esser T.K., Böhning J., Fremdling P., Agasid M.T., Costin A., Fort K. et al. (2022) Mass-selective and ice-free electron cryomicroscopy protein sample preparation via native electrospray ion-beam deposition. PNAS Nexus 1, pgac153 10.1093/pnasnexus/pgac153 36714824 PMC9802471

[26] Westphall M.S., Lee K.W., Salome A.Z., Lodge J.M., Grant T. and Coon J.J. (2022) Three-dimensional structure determination of protein complexes using matrix-landing mass spectrometry. Nat. Commun. 13, 2276 10.1038/s41467-022-29964-4 35478194 PMC9046196

[27] Niebling S., Veith K., Vollmer B., Lizarrondo J., Burastero O., Schiller J. et al. (2022) Biophysical screening pipeline for Cryo-EM grid preparation of membrane proteins. Front. Mol. Biosci. 9, 882288 10.3389/fmolb.2022.882288 35813810 PMC9259969

[28] Olinares P.D.B., Kang J.Y., Llewellyn E., Chiu C., Chen J., Malone B. et al. (2021) Native mass spectrometry-based screening for optimal sample preparation in single-particle Cryo-EM. Structure 29, 186.e186–195.e186 10.1016/j.str.2020.11.001 33217329 PMC7867593

[29] Pyle E., Kalli A.C., Amillis S., Hall Z., Lau A.M., Hanyaloglu A.C. et al. (2018) Structural lipids enable the formation of functional oligomers of the eukaryotic purine symporter UapA. Cell Chem. Biol. 25, 840.e844–848.e844 10.1016/j.chembiol.2018.03.011 29681524 PMC6058078

[30] Hoi K.K., Bada Juarez J.F., Judge P.J., Yen H.Y., Wu D., Vinals J. et al. (2021) Detergent-free lipodisq nanoparticles facilitate high-resolution mass spectrometry of folded integral membrane proteins. Nano Lett. 21, 2824–2831 10.1021/acs.nanolett.0c04911 33787280 PMC8050825

[31] Liu R., Xia S. and Li H. (2022) Native top-down mass spectrometry for higher-order structural characterization of proteins and complexes. Mass Spectrom. Rev. e21793 10.1002/mas.21793 35757976

[32] Cong X., Patrick J.W., Liu Y., Liang X., Liu W. and Laganowsky A. (2022) Investigation of protein-lipid interactions using native mass spectrometry. Methods Mol. Biol. 2349, 41–64 10.1007/978-1-0716-1585-0_3 34718990

[33] Keener J.E., Zhang G. and Marty M.T. (2021) Native mass spectrometry of membrane proteins. Anal. Chem. 93, 583–597 10.1021/acs.analchem.0c04342 33115234 PMC7855921

[34] Anandan A. and Vrielink A. (2016) Detergents in membrane protein purification and crystallisation. Adv. Exp. Med. Biol. 922, 13–28 10.1007/978-3-319-35072-1_2 27553232

[35] Seddon A.M., Curnow P. and Booth P.J. (2004) Membrane proteins, lipids and detergents: not just a soap opera. Biochim. Biophys. Acta 1666, 105–117 10.1016/j.bbamem.2004.04.011 15519311

[36] Kurauskas V., Hessel A., Ma P., Lunetti P., Weinhäupl K., Imbert L. et al. (2018) How detergent impacts membrane proteins: atomic-level views of mitochondrial carriers in dodecylphosphocholine. J. Phys. Chem. Lett. 9, 933–938 10.1021/acs.jpclett.8b00269 29397729 PMC5834942

[37] Bordag N. and Keller S. (2010) α-Helical transmembrane peptides: A “Divide and Conquer” approach to membrane proteins. Chem. Phys. Lipids 163, 1–26 10.1016/j.chemphyslip.2009.07.009 19682979

[38] Kotov V., Bartels K., Veith K., Josts I., Subhramanyam U.K.T., Günther C. et al. (2019) High-throughput stability screening for detergent-solubilized membrane proteins. Sci. Rep. 9, 10379 10.1038/s41598-019-46686-8 31316088 PMC6637136

[39] Landreh M., Costeira-Paulo J., Gault J., Marklund E.G. and Robinson C.V. (2017) Effects of detergent micelles on lipid binding to proteins in electrospray ionization mass spectrometry. Anal. Chem. 89, 7425–7430 10.1021/acs.analchem.7b00922 28627869 PMC5559180

[40] Reading E., Walton T.A., Liko I., Marty M.T., Laganowsky A., Rees D.C. et al. (2015) The effect of detergent, temperature, and lipid on the oligomeric state of MscL constructs: insights from mass spectrometry. Chem. Biol. 22, 593–603 10.1016/j.chembiol.2015.04.016 26000747 PMC6585436

[41] Bolla J.R., Corey R.A., Sahin C., Gault J., Hummer A., Hopper J.T.S. et al. (2020) A mass-spectrometry-based approach to distinguish annular and specific lipid binding to membrane proteins. Angew. Chem. Int. Ed. Engl. 59, 3523–3528 10.1002/anie.201914411 31886601 PMC7065234

[42] Reading E., Liko I., Allison T.M., Benesch J.L., Laganowsky A. and Robinson C.V. (2015) The role of the detergent micelle in preserving the structure of membrane proteins in the gas phase. Angew. Chem. Int. Ed. 54, 4577–4581 10.1002/anie.201411622 25693501

[43] Urner L.H., Liko I., Yen H.Y., Hoi K.K., Bolla J.R., Gault J. et al. (2020) Modular detergents tailor the purification and structural analysis of membrane proteins including G-protein coupled receptors. Nat. Commun. 11, 564 10.1038/s41467-020-14424-8 31992701 PMC6987200

[44] Urner L.H., Schulze M., Maier Y.B., Hoffmann W., Warnke S., Liko I. et al. (2020) A new azobenzene-based design strategy for detergents in membrane protein research. Chem. Sci. 11, 3538–3546 10.1039/D0SC01022G 34109026 PMC8152689

[45] Marty M.T., Hoi K.K. and Robinson C.V. (2016) Interfacing membrane mimetics with mass spectrometry. Acc. Chem. Res. 49, 2459–2467 10.1021/acs.accounts.6b00379 27736086 PMC5328410

[46] Hopper J.T.S., Yu Y.T.-C., Li D., Raymond A., Bostock M., Liko I. et al. (2013) Detergent-free mass spectrometry of membrane protein complexes. Nat. Methods 10, 1206–1208 10.1038/nmeth.2691 24122040 PMC3868940

[47] Knowles T.J., Finka R., Smith C., Lin Y.-P., Dafforn T. and Overduin M. (2009) Membrane proteins solubilized intact in lipid containing nanoparticles bounded by styrene maleic acid copolymer. J. Am. Chem. Soc. 131, 7484–7485 10.1021/ja810046q 19449872

[48] Oluwole A.O., Danielczak B., Meister A., Babalola J.O., Vargas C. and Keller S. (2017) Solubilization of membrane proteins into functional lipid-bilayer nanodiscs using a diisobutylene/maleic acid copolymer. Angew. Chem. Int. Ed. Engl. 56, 1919–1924 10.1002/anie.201610778 28079955 PMC5299484

[49] Hellwig N., Peetz O., Ahdash Z., Tascón I., Booth P.J., Mikusevic V. et al. (2018) Native mass spectrometry goes more native: investigation of membrane protein complexes directly from SMALPs. Chem. Commun. 54, 13702–13705 10.1039/C8CC06284F PMC6289172 30452022

[50] Fantin S.M., Parson K.F., Yadav P., Juliano B., Li G.C., Sanders C.R. et al. (2021) Ion mobility–mass spectrometry reveals the role of peripheral myelin protein dimers in peripheral neuropathy. Proc. Natl. Acad. Sci. 118, e2015331118 10.1073/pnas.2015331118 33893233 PMC8092585

[51] Keener J.E., Jayasekera H.S. and Marty M.T. (2022) Investigating the lipid selectivity of membrane proteins in heterogeneous nanodiscs. Anal. Chem. 94, 8497–8505 10.1021/acs.analchem.2c01488 35621361 PMC9197995

[52] Kostelic M.M., Zak C.K., Jayasekera H.S. and Marty M.T. (2021) Assembly of model membrane nanodiscs for native mass spectrometry. Anal. Chem. 93, 5972–5979 10.1021/acs.analchem.1c00735 33797873

[53] Keener J.E., Zambrano D.E., Zhang G., Zak C.K., Reid D.J., Deodhar B.S. et al. (2019) Chemical additives enable native mass spectrometry measurement of membrane protein oligomeric state within intact nanodiscs. J. Am. Chem. Soc. 141, 1054–1061 10.1021/jacs.8b11529 30586296 PMC6475481

[54] Chen S., Getter T., Salom D., Wu D., Quetschlich D., Chorev D.S. et al. (2022) Capturing a rhodopsin receptor signalling cascade across a native membrane. Nature 604, 384–390 10.1038/s41586-022-04547-x 35388214 PMC9007743

[55] Chorev D.S., Baker L.A., Wu D., Beilsten-Edmands V., Rouse S.L., Zeev-Ben-Mordehai T. et al. (2018) Protein assemblies ejected directly from native membranes yield complexes for mass spectrometry. Science 362, 829–834 10.1126/science.aau0976 30442809 PMC6522346

[56] Harvey S.R., O'Neale C., Schey K.L. and Wysocki V.H. (2022) Native mass spectrometry and surface induced dissociation provide insight into the post-translational modifications of tetrameric AQP0 isolated from bovine eye lens. Anal. Chem. 94, 1515–1519 10.1021/acs.analchem.1c04322 35015511 PMC9161558

[57] Yang Y., Liu J., Clarke B.R., Seidel L., Bolla J.R., Ward P.N. et al. (2021) The molecular basis of regulation of bacterial capsule assembly by Wzc. Nat. Commun. 12, 4349 10.1038/s41467-021-24652-1 34272394 PMC8285477

[58] Ro S.Y., Schachner L.F., Koo C.W., Purohit R., Remis J.P., Kenney G.E. et al. (2019) Native top-down mass spectrometry provides insights into the copper centers of membrane-bound methane monooxygenase. Nat. Commun. 10, 2675 10.1038/s41467-019-10590-6 31209220 PMC6572826

[59] Fantin S.M., Parson K.F., Yadav P., Juliano B., Li G.C., Sanders C.R. et al. (2021) Ion mobility-mass spectrometry reveals the role of peripheral myelin protein dimers in peripheral neuropathy. Proc. Natl. Acad. Sci. U.S.A. 118, e2015331118 10.1073/pnas.2015331118 33893233 PMC8092585

[60] Abbas Y.M., Wu D., Bueler S.A., Robinson C.V. and Rubinstein J.L. (2020) Structure of V-ATPase from the mammalian brain. Science 367, 1240–1246 10.1126/science.aaz2924 32165585 PMC7324285

[61] Landreh M., Marty M.T., Gault J. and Robinson C.V. (2016) A sliding selectivity scale for lipid binding to membrane proteins. Curr. Opin. Struct. Biol. 39, 54–60 10.1016/j.sbi.2016.04.005 27155089 PMC5287393

[62] Laganowsky A., Reading E., Allison T.M., Ulmschneider M.B., Degiacomi M.T., Baldwin A.J. et al. (2014) Membrane proteins bind lipids selectively to modulate their structure and function. Nature 510, 172–175 10.1038/nature13419 24899312 PMC4087533

[63] Oluwole A.O., Corey R.A., Brown C.M., Hernández-Rocamora V.M., Stansfeld P.J., Vollmer W. et al. (2022) Peptidoglycan biosynthesis is driven by lipid transfer along enzyme-substrate affinity gradients. Nat. Commun. 13, 1–12 10.1038/s41467-022-29836-x 35477938 PMC9046198

[64] Patil D.N., Singh S., Laboute T., Strutzenberg T.S., Qiu X., Wu D. et al. (2022) Cryo-EM structure of human GPR158 receptor coupled to the RGS7-Gβ5 signaling complex. Science 375, 86–91 10.1126/science.abl4732 34793198 PMC8926151

[65] Webby M.N., Oluwole A.O., Pedebos C., Inns P.G., Olerinyova A., Prakaash D. et al. (2022) Lipids mediate supramolecular outer membrane protein assembly in bacteria. Sci. Adv. 8, eadc9566 10.1126/sciadv.adc9566 36322653 PMC9629720

[66] Bolla J.R., Howes A.C., Fiorentino F. and Robinson C.V. (2020) Assembly and regulation of the chlorhexidine-specific efflux pump AceI. Proc. Natl. Acad. Sci. 117, 17011–17018 10.1073/pnas.2003271117 32636271 PMC7382281

[67] Fiorentino F., Sauer J.B., Qiu X., Corey R.A., Cassidy C.K., Mynors-Wallis B. et al. (2021) Dynamics of an LPS translocon induced by substrate and an antimicrobial peptide. Nat. Chem. Biol. 17, 187–195 10.1038/s41589-020-00694-2 33199913 PMC7116625

[68] Kaur H., Jakob R.P., Marzinek J.K., Green R., Imai Y., Bolla J.R. et al. (2021) The antibiotic darobactin mimics a β-strand to inhibit outer membrane insertase. Nature 593, 125–129 10.1038/s41586-021-03455-w 33854236

[69] Petroff J.T., Tong I.I., Chen A., Dekoster L.J., Khan G.T., Abramson F. et al. (2020) Charge reduction of membrane proteins in native mass spectrometry using alkali metal acetate salts. Anal. Chem. 92, 6622–6630 10.1021/acs.analchem.0c00454 32250604 PMC7275249

[70] Hernández H. and Robinson C.V. (2007) Determining the stoichiometry and interactions of macromolecular assemblies from mass spectrometry. Nat. Protoc. 2, 715–726 10.1038/nprot.2007.73 17406634

[71] Xia Z. and Williams E.R. (2019) Effect of droplet lifetime on where ions are formed in electrospray ionization. Analyst 144, 237–248 10.1039/C8AN01824C 30488074

[72] Agasid M.T., Sørensen L., Urner L.H., Yan J. and Robinson C.V. (2021) The Effects of Sodium Ions on Ligand Binding and Conformational States of G Protein-Coupled Receptors—Insights from Mass Spectrometry. J. Am. Chem. Soc. 143, 4085–4089 10.1021/jacs.0c11837 33711230 PMC7995251

[73] Yen H.-Y., Liko I., Song W., Kapoor P., Almeida F., Toporowska J. et al. (2022) Mass spectrometry captures biased signalling and allosteric modulation of a G-protein-coupled receptor. Nat. Chem. 14, 1375–1382 10.1038/s41557-022-01041-9 36357787 PMC9758051

[74] Gray D.A., White J.B.R., Oluwole A.O., Rath P., Glenwright A.J., Mazur A. et al. (2021) Insights into SusCD-mediated glycan import by a prominent gut symbiont. Nat. Commun. 12, 44 10.1038/s41467-020-20285-y 33398001 PMC7782687

[75] Bolla J.R., Sauer J.B., Wu D., Mehmood S., Allison T.M. and Robinson C.V. (2018) Direct observation of the influence of cardiolipin and antibiotics on lipid II binding to MurJ. Nat. Chem. 10, 363–371 10.1038/nchem.2919 29461535 PMC5912511

[76] Chorev D.S., Tang H., Rouse S.L., Bolla J.R., von Kügelgen A., Baker L.A. et al. (2020) The use of sonicated lipid vesicles for mass spectrometry of membrane protein complexes. Nat. Protoc. 15, 1690–1706 10.1038/s41596-020-0303-y 32238951 PMC7305028

[77] Gupta K., Li J., Liko I., Gault J., Bechara C., Wu D. et al. (2018) Identifying key membrane protein lipid interactions using mass spectrometry. Nat. Protoc. 13, 1106–1120 10.1038/nprot.2018.014 29700483 PMC6049616

[78] Gault J., Liko I., Landreh M., Shutin D., Bolla J.R., Jefferies D. et al. (2020) Combining native and ‘omics’ mass spectrometry to identify endogenous ligands bound to membrane proteins. Nat. Methods 17, 505–508 10.1038/s41592-020-0821-0 32371966 PMC7332344

[79] Jin R., He S., Black K.A., Clarke O.B., Wu D., Bolla J.R. et al. (2022) Ion currents through Kir potassium channels are gated by anionic lipids. Nat. Commun. 13, 490 10.1038/s41467-022-28148-4 35079013 PMC8789855

[80] Khera R., Mehdipour A.R., Bolla J.R., Kahnt J., Welsch S., Ermler U. et al. (2022) Cryo-EM structures of pentameric autoinducer-2 exporter from *Escherichia coli* reveal its transport mechanism. EMBO J. 41, e109990 10.15252/embj.2021109990 35698912 PMC9475539

[81] Sicoli G., Konijnenberg A., Guérin J., Hessmann S., Del Nero E., Hernandez-Alba O. et al. (2022) Large-scale conformational changes of FhaC provide insights into the two-partner secretion mechanism. Front. Mol. Biosci. 9, 950871 10.3389/fmolb.2022.950871 35936790 PMC9355242

[82] Kjølbye L.R., Sørensen L., Yan J., Berglund N.A., Ferkinghoff-Borg J., Robinson C.V. et al. (2022) Lipid modulation of a class B GPCR: elucidating the modulatory role of PI(4,5)P2 Lipids. J. Chem. Inf. Model. 62, 6788–6802 10.1021/acs.jcim.2c00635 36036575

[83] Yen H.Y., Abramsson M.L., Agasid M.T., Lama D., Gault J., Liko I. et al. (2022) Electrospray ionization of native membrane proteins proceeds via a charge equilibration step. RSC Adv. 12, 9671–9680 10.1039/D2RA01282K 35424940 PMC8972943

[84] Matsuoka R., Fudim R., Jung S., Zhang C., Bazzone A., Chatzikyriakidou Y. et al. (2022) Structure, mechanism and lipid-mediated remodeling of the mammalian Na(+)/H(+) exchanger NHA2. Nat. Struct. Mol. Biol. 29, 108–120 10.1038/s41594-022-00738-2 35173351 PMC8850199

[85] Yen H.-Y., Liko I., Song W., Kapoor P., Almeida F., Toporowska J. et al. (2022) Mass spectrometry captures biased signalling and allosteric modulation of a G-protein-coupled receptor. Nat. Chem. 14, 1375–1382 10.1038/s41557-022-01041-9 36357787 PMC9758051

[86] van den Berg B., Pedebos C., Bolla J.R., Robinson C.V., Baslé A. and Khalid S. (2021) Structural basis for silicic acid uptake by higher plants. J. Mol. Biol. 433, 167226 10.1016/j.jmb.2021.167226 34487790

[87] Zhao J., Hellwig N., Djahanschiri B., Khera R., Morgner N., Ebersberger I. et al. (2022) Assembly and functional role of PACE transporter PA2880 from *Pseudomonas aeruginosa*. Microbiol. Spectrum 10, e01453–e01421 10.1128/spectrum.01453-21 PMC9045395 35377188

[88] Guffick C., Hsieh P.-Y., Ali A., Shi W., Howard J., Chinthapalli D.K. et al. (2022) Drug-dependent inhibition of nucleotide hydrolysis in the heterodimeric ABC multidrug transporter PatAB from Streptococcus pneumoniae. FEBS J. 289, 3770–3788 10.1111/febs.16366 35066976 PMC9541285

[89] Schmidpeter P.A.M., Wu D., Rheinberger J., Riegelhaupt P.M., Tang H., Robinson C.V. et al. (2022) Anionic lipids unlock the gates of select ion channels in the pacemaker family. Nat. Struct. Mol. Biol. 29, 1092–1100 10.1038/s41594-022-00851-2 36352139 PMC10022520

[90] Zhu Y., Schrecke S., Tang S., Odenkirk M.T., Walker T., Stover L. et al. (2022) Cupric Ions Selectively Modulate TRAAK–Phosphatidylserine Interactions. J. Am. Chem. Soc. 144, 7048–7053 10.1021/jacs.2c00612 35421309 PMC9136672

[91] Grison C.M., Lambey P., Jeannot S., Del Nero E., Fontanel S., Peysson F. et al. (2021) Molecular insights into mechanisms of GPCR hijacking by *Staphylococcus aureus*. Proc. Natl. Acad. Sci. 118, e2108856118 10.1073/pnas.2108856118 34663701 PMC8545443

[92] Qiao P., Schrecke S., Lyu J., Zhu Y., Zhang T., Benavides A. et al. (2021) Insight into the Phospholipid-binding preferences of Kir3.4. Biochemistry 60, 3813–3821 10.1021/acs.biochem.1c00615 34846128 PMC9569133

[93] Cater R.J., Chua G.L., Erramilli S.K., Keener J.E., Choy B.C., Tokarz P. et al. (2021) Structural basis of omega-3 fatty acid transport across the blood–brain barrier. Nature 595, 315–319 10.1038/s41586-021-03650-9 34135507 PMC8266758

[94] Moysa A., Steczkiewicz K., Niedzialek D., Hammerschmid D., Zhukova L., Sobott F. et al. (2021) A model of full-length RAGE in complex with S100B. Structure 29, 989.e1006–1002.e1006 10.1016/j.str.2021.04.002 33887170

[95] Norris C.E., Keener J.E., Perera S.M.D.C., Weerasinghe N., Fried S.D.E., Resager W.C. et al. (2021) Native mass spectrometry reveals the simultaneous binding of lipids and zinc to rhodopsin. Int. J. Mass Spectrom. 460, 116477 10.1016/j.ijms.2020.116477 33281496 PMC7709953

[96] Stefan E., Obexer R., Hofmann S., Vu Huu K., Huang Y., Morgner N. et al. (2021) *De novo* macrocyclic peptides dissect energy coupling of a heterodimeric ABC transporter by multimode allosteric inhibition. eLife 10, e67732 10.7554/eLife.67732 33929325 PMC8116058

[97] Housden N.G., Webby M.N., Lowe E.D., El-Baba T.J., Kaminska R., Redfield C. et al. (2021) Toxin import through the antibiotic efflux channel TolC. Nat. Commun. 12, 4625 10.1038/s41467-021-24930-y 34330923 PMC8324772

[98] Vorobieva A.A., White P., Liang B., Horne J.E., Bera A.K., Chow C.M. et al. (2021) *De novo* design of transmembrane β-barrels. Science 371, eabc8182 10.1126/science.abc8182 33602829 PMC8064278

[99] Reading E., Ahdash Z., Fais C., Ricci V., Wang-Kan X., Grimsey E. et al. (2020) Perturbed structural dynamics underlie inhibition and altered efflux of the multidrug resistance pump AcrB. Nat. Commun. 11, 5565 10.1038/s41467-020-19397-2 33149158 PMC7642415

[100] Urner L.H., Schulze M., Maier Y.B., Hoffmann W., Warnke S., Liko I. et al. (2020) A new azobenzene-based design strategy for detergents in membrane protein research. Chem. Sci. 11, 3538–3546 10.1039/D0SC01022G 34109026 PMC8152689

[101] Neviani V., van Deventer S., Wörner T.P., Xenaki K.T., van de Waterbeemd M., Rodenburg R.N.P. et al. (2020) Site-specific functionality and tryptophan mimicry of lipidation in tetraspanin CD9. FEBS J. 287, 5323–5344 10.1111/febs.15295 32181977 PMC7818406

[102] Zhu G., Zeng H., Zhang S., Juli J., Pang X., Hoffmann J. et al. (2020) A 3.3 Å-resolution structure of hyperthermophilic respiratory complex III reveals the mechanism of its thermal stability. Angew. Chem. Int. Ed. Engl. 59, 343–351 10.1002/anie.201911554 31778296 PMC7004027

[103] Wang L., Qian H., Nian Y., Han Y., Ren Z., Zhang H. et al. (2020) Structure and mechanism of human diacylglycerol O-acyltransferase 1. Nature 581, 329–332 10.1038/s41586-020-2280-2 32433610 PMC7255049

[104] Tan Y.Z., Rodrigues J., Keener J.E., Zheng R.B., Brunton R., Kloss B. et al. (2020) Cryo-EM structure of arabinosyltransferase EmbB from Mycobacterium smegmatis. Nat. Commun. 11, 3396 10.1038/s41467-020-17202-8 32636380 PMC7341804

[105] Behrens H.M., Lowe E.D., Gault J., Housden N.G., Kaminska R., Weber T.M. et al. (2020) Pyocin S5 Import into *Pseudomonas aeruginosa* Reveals a Generic Mode of Bacteriocin Transport. mBio 11, e03230–19 10.1128/mBio.03230-19 32156826 PMC7064778

[106] McDowell M.A., Heimes M., Fiorentino F., Mehmood S., Farkas Á., Coy-Vergara J. et al. (2020) Structural basis of tail-anchored membrane protein biogenesis by the GET Insertase complex. Mol. Cell 80, 72.e77–86.e77 10.1016/j.molcel.2020.08.012 32910895

[107] Zeng H., Zhu G., Zhang S., Li X., Martin J., Morgner N. et al. (2020) Isolated Heme A Synthase from Aquifex aeolicus Is a Trimer. mBio 11, e02615–e02619 10.1128/mBio.02615-19 32605991 PMC7327177

[108] Schrecke S., Zhu Y., McCabe J.W., Bartz M., Packianathan C., Zhao M. et al. (2021) Selective regulation of human TRAAK channels by biologically active phospholipids. Nat. Chem. Biol. 17, 89–95 10.1038/s41589-020-00659-5 32989299 PMC7746637

[109] Kono A. and Spalding M.H. (2020) LCI1, a Chlamydomonas reinhardtii plasma membrane protein, functions in active CO_2_ uptake under low CO_2_. Plant J. 102, 1127–1141 10.1111/tpj.14761 32248584

[110] Winkelmann I., Matsuoka R., Meier P.F., Shutin D., Zhang C., Orellana L. et al. (2020) Structure and elevator mechanism of the mammalian sodium/proton exchanger NHE9. EMBO J. 39, e105908 10.15252/embj.2020105908 33118634 PMC7737618

[111] Jansen K.B., Inns P.G., Housden N.G., Hopper J.T.S., Kaminska R., Lee S. et al. (2020) Bifurcated binding of the OmpF receptor underpins import of the bacteriocin colicin N into *Escherichia coli*. J. Biol. Chem. 295, 9147–9156 10.1074/jbc.RA120.013508 32398259 PMC7335789

